# Suprapubic Cystotomy

**Published:** 1891-11

**Authors:** R. W. Stewart

**Affiliations:** Pittsburg, Pa.


### SUPRAPUBIC CYSTOTOMY.*

BY R. W. STEWART, M. D., PITTSBURG, PA.

The following cases operated on by myself during the present year, and given in the order of their occurrence, will serve to show some of the conditions for which this operation is indicated, and also serve as a basis for further remarks on the operation:

CASE I.—This patient was under the care of Dr. Grube, who has kindly furnished me with the following notes of the case:

February 10th, 1891. J. O., age 32, furnaceman. Patient says that about six months ago he first noticed difficulty in urination, with pain in bladder and penis. This gradually passed into chronic cystitis, accompanied by pain in legs and partial paraplegia. He was treated for cystitis at Mercy Hospital. The bladder is extremely irritable, and holds scarcely an ounce, and

* Read before Allegheny County Medical Society, August 18, 1891.

as the slightest distension causes intense pain, it is impossible for him to sleep longer than half an hour at a time ; consequently he is greatly reduced in strength. The stomach is irritable, and digestion impaired ; patient living almost entirely on milk. The prostate gland is slightly enlarged, and is nodular, leading to the suspicion that it is tubercular. Patient has a brother who has pulmonary tuberculosis, and he himself has had a cough for several years, though his lungs are not perceptibly tubercular. Urine contains large quantities of muco-pus. Microscope shows pus cells, caseous flakes and débris. As patient was under Dr. Stewart's care at Mercy Hospital, I have asked him to see patient, and we have decided upon suprapubic cystotomy.

February 14th. Dr. Stewart operated as above, assisted by Drs. Ward, Patterson, Emmerling and myself. As the bladder would not bear distension by fluid, the fundus was pushed up into wound by point of sound. A papillomatous growth was removed from near entrance of left ureter—about a teaspoonful of scrapings in all. Wound closed and bladder drained by single large drainage tube ; directed daily washing out of bladder with boro-salicylic acid solution.

February 20th. Patient has been given great relief from irritability of bladder, and is grateful accordingly. Urine still muco-purulent ; general condition, bad.

March 10th. Wound has healed nicely around drainage tube, and patient manages drainage and washing out of the bladder himself.

April 1st. No improvement in character of urine, and patient losing ground steadily. There are occasional discharges of caseous-looking pus from urethra, which evidently comes from the prostate. Tubercles have made their appearance in the cicatricial tissue about the drainage tube.

The further progress of this case was a gradual decline, until he died about the middle of May.

CASE II.—Daniel R., age 54. About eight years ago he had several attacks apparently of renal colic, occurring at intervals of two months. After this there was a period of quiescence until about eighteen months ago, when he complained of frequency in passing water, the termination of the act being associated with pain, which was referred to the end of the penis. Exertion of any sort aggravated the trouble, while on the contrary, rest in the recumbent position diminished it. So frequent had become the calls to urinate, and so difficult to restrain the desire, that it was necessary to wear a urinal. For about a year the patient was unable to pursue his vocation of machinist. He was referred to me for treatment by Dr. Ward.

Owing to the extreme sensitiveness of the patient and the irritability of the bladder, an examination without the aid of an anæsthetic was a matter of considerable difficulty, and required the utmost tact and delicacy. A diagnosis of vesical calculus was made, and the patient sent to Mercy Hospital for operation. Accordingly, on March 15th the patient being anæsthetized, a rectal bag was inserted and distended with eight ounces of water. The suprapubic operation was then performed, and three calculi lying side by side were removed. A drainage tube was inserted in the bladder and the wound partially closed with three silver sutures. A loose gauze dressing was applied over all.

The condition of the patient after operation was satisfactory, and was devoid of constitutional disturbance. He left the hospital on the seventeenth day following the operation. A fistulous opening still communicated with the bladder, which was somewhat slow in healing, but eventually it closed, and at this date patient is in good health, has full control of his urine and is free from pain.

CASE III.—Louis M., age 34, a butcher by occupation. On the evening of May 28th, he stepped on a coal-hole, the lid of which turned and he fell, the edge of the lid striking him on the perineum. He was able to walk a short distance, and then took a carriage home. On the following morning he was suffering from retention of urine, and Dr. Speer was called to see him. With a soft catheter he withdrew a quantity of bloody urine. On the evening of the same day I was called in consultation, the doctor being unable to withdraw his urine. At that time the bladder was distended, perineum tender, swollen, and much discolored, the discoloration extending to the scrotum. A diagnosis of rupture of the urethra at the triangular ligament was made, and with the assistance of Drs. Speer, Christler, and McKibben, the patient being anæsthetized, I opened the perineum freely in several places, through which a small quantity of bloody urine escaped. A complete rupture of the urethra was discovered. Owing to the extravasation, the tissues were so altered in appearance that it was impossible to distinguish the vesical end of the torn urethra; after a patient attempt I abandoned the search for it, and ordered his removal to Mercy Hospital. He did not enter the hospital on the following day, and as he was still suffering from retention it was necessary to asperate his bladder in the morning and evening. On the following morning he entered the hospital, and I operated on him again. At this time the patient's temperature was 103° F., and his general condition was bad. Being again unable to find the vesical end of the urethra, I opened the distended bladder above the pubes, the incision in the bladder being just sufficient to admit a steel sound, with which I performed retrograde catheterism. The sound, after passing from within outwards through the prostatic urethra, was made to project through the perineal opening. While in this situation a stout rubber tube was fitted on the projecting conical extremity of the sound, which, together with the tube, was withdrawn into the bladder, and the sound disengaged from the tube. The sound was then passed from before backwards through the pendulous urethra, the extremity again presenting through the perineal opening. On this was fitted the end of the rubber tube which projected from the perineal opening, and the sound carrying with it the tube was withdrawn. By this manœuvre a tube was inserted in the whole length of the urethra, one end being in the bladder and the other projecting from the external meatus, the central portion bridging over the torn ends of the urethra, which were separated by an interval of about three-quarters of an inch. Displacement of the tube was prevented by pinning it to the prepuce. The patient's condition improved at once ; his temperature was normal on the third day. The urine drained through the tube. A slight leakage escaped through suprapubic opening. On the eighth day the tube was removed, and the patient left the hospital on the twelfth day, since which time no urine has passed by suprapubic opening. A No. 26 French sound has been passed at intervals since that date. At present the sound is passed once every two weeks to prevent the formation of a stricture at site of injury, and except for this inconvenience the patient is as well as he ever was.

CASE IV.—A. M. W., age 21. Ten years ago this patient was suddenly attacked with a desire to urinate frequently, which he attributed to holding his urine too long. This condition has persisted without intermission during the past ten years, passing water every twenty to forty minutes, night and day, the act being associated with violent tenesmus, and, at times, excruciating pain. The constant straining has produced a marked prolapse of the rectum, which protrudes during the act to the extent of about five inches.

Three years after the onset of this attack he became subject to epileptic seizures, which would occur about once a month, and in some manner seemed to be associated with an exacerbation of his vesical trouble. I may anticipate by saying that since the latter has been relieved the convulsions have ceased.

During the ten years he has suffered he has tried various forms of treatment in hospitals and out of them, under regulars and irregulars, besides his attempts at self-cure with the aid of patent medicines, all of which, to use his own language, did him no good, and he was waiting to die. Finally Dr. Buchanan sent him to Mercy Hospital, and he was transferred to me. The case was and still is something of a puzzle. The sound failed to shed any light on the subject, the cystoscope was also used, but nothing abnormal could be detected; external perineal urethrotomy performed, and a digital examination of the interior of the bladder was made by Dr. Buchanan and myself, but nothing abnormal, further than a dilatation of the opening of the right ureter could be detected. Into this opening I readily inserted the beak of Thompson's seacher, which passed, without obstruction, along the ureter until it must have reached the pelvis of the kidney. In this situation the searcher could be readily turned in any direction, showing that the ureter was much dilated. While the searcher was in this situation the descent of the liver in inspiration could be readily felt pressing against the extremity of the instrument. The ureter contained about an ounce of apparently healty urine, which escaped along the hollow instrument. A drainage tube was inserted in the perineal opening, and the bladder drained by this means for ten days. During this period the patient had comparative comfort, and for the first time in ten years he was able to sleep a few hours at a time. After the tube was withdrawn on the tenth day, the perineal opening closed, and the patient relapsed into his previous miserable condition.

The results of all these examinations showed that we were no nearer the solution of the cause of this trouble. Whether the dilated ureter was the cause, or the result of the frequent urination, we were unable to determine; one thing, however, was apparent, that drainage of the bladder relieved the symptoms, and I therefore decided to establish permanent drainage.

In this operation I was again assisted by Dr. Buchanan. A specially contrived sound, having a greater curve than the ordinary sound, and a tip on it over which a tube could be readily fitted, was used. The extremity of the instrument could be felt just above the pubes, an incision was made over it, and the instrument presented itself in the wound. A tube was inserted over the tip of the instrument, which was withdrawn, leaving the tube in the bladder, and a permanent drainage was now established. The patient, in a short time, was able to manage the tube himself, taking it out twice daily, and washing the bladder with a weak bi-chloride solution, the free extremity of the tube fitting into a urinal by day, and at night connected with a long tube which carries the urine to a vessel placed at the bedside. When last seen he had gained in flesh, could sleep without interruption, and for the first time in his life he was making arrangements to earn a living for himself.

The operation of suprapubic cystotomy has, within the past few years, attracted considerable attention, and is now looked on with more favor than at any previous period. Some have gone so far as to condemn entirely the perineal route to the bladder, and assert that the suprapubic route should be used exclusively ; but that this is going too far will be evident to any one who will give the subject a little attention. For temporary drainage and for digital exploration, the perineal method of opening the bladder is undoubtedly the simplest and the safest. On the other hand, the suprapubic method is, in the majority of cases, to be preferred for the removal of calculi too large to be crushed ; also for the removal of tumors, with the possible exception of prostatic growths, and for the establishment of permanant drainage. This operation has been hedged around with so many precautions and imaginary dangers that what is really a very simple operation appears to the uninitiated to be one of great magnitude.

Elaborate dissections have been made to show the relationship of the vesico-parietal peritoneal reflection to the operation, and the benefits of rectal and vesical distension have been urged. The dangers of urinary extravasation and hæmorrhage have been pointed out, and the advantages of Trendelenberg's position dilated upon. Regarding the much-talked-of peritoneum : In none of the cases that I have recorded was it seen during the operation, and in only one of them was a rectal bag used. While vesical distension was resorted to in none, though present as an accidental occurrence in case three, the advantages of both of these have, in my opinion, been more than counterbalanced by the risks incurred from over-distension in their use. A longitudinal incision was used, keeping close to the upper border of the symphysis pubis, and the bladder opened on the tip of a well-curved sound, the finger being kept at the same time in the upper border of the wound to prevent displacement downwards of the peritoneum and intestines. A pair of forceps was next insinuated alongside the sound, into the bladder, and expanded so as to tear the vesical opening to the extent desired. Hæmorrhage was not troublesome in any case. No attempt was made to suture the vesical wound, nor would I recommend that it be attempted, unless the opening was very large. In three of the cases the abdominal wound was partially closed with silver sutures ; but in each of these cases the wound reopened on removal of the sutures, so that in the future I will dispense with their use. I would recommend, however, that the incision, both in the abdominal wall and bladder, be limited to the smallest extent consistent with the requirements for operating within the bladder.

No constitutional disturbance was produced by the operation in any case, no extravasation of urine occurred, and the after-treatment consisted of frequent renewal of the dressings and washing out of the bladder with a mild antiseptic solution.

---

EHRLICH'S TEST IN TYPHOID FEVER.—This test, which has been known for a number of years, has till recently been regarded by many rather as a medical curiosity than as of diagnostic value. Dr. C. E. Simon, of the Johns Hopkins Hospital, has recently shown that by carefully following the precise directions for its use valuable information may be derived. The test consists of two solutions : 1. A saturated solution of sulphanilic acid in five per cent. hydrochloric acid. 2. A five per cent solution of sodium nitrate. These are to be mixed, just before use, in the proportion of 40 c.c. of (1) to 1 c.c. of (2). If this mixture be added to urine from a case of typhoid fever, the further addition of ammonia will produce a play of colors varying from an eosine rose to a deep garnet red. The best method of applying the test is to take a few centimetres of urine in a test-tube, adding an equal quantity of the sulphanilic acid mixture, and shake thoroughly ; 1 c.c. of ammonia is then run carefully down the side of the tube. At the junction of the two liquids there will be observed a ring of the characteristic color, which is produced in scarcely any other disease than typhoid fever. Dr. Simon's conclusions may be thus summarized : 1. The reaction may be obtained in typhoid fever from the fifth to the twenty-second day of the disease. 2. Its absence from the fifth to the ninth day indicates a very mild attack, save in children, although this rule is not absolute one. 3. As it occurs previous to the appearance of the rash, it is a very useful aid in the diagnosis of typhoid fever.—*Therapeutic Gazette.*

